# The molecular determinants governing the immunogenicity of Japanese encephalitis live attenuated vaccines

**DOI:** 10.1038/sigtrans.2017.5

**Published:** 2017-03-24

**Authors:** Yuhua Li, Yin Fu, Xinyu Liu, Huiqiang Yang, Yongxin Yu, Lili Jia, Xuguang Li, Aaron Farnsworth, Junzhi Wang

**Affiliations:** 1 National Institutes for Food and Drug Control, Beijing, China; 2 State Key Laboratory of Biotherapy and Cancer Center, West China Hospital, Sichuan University, Collaborative Innovation Center for Biotherapy; 3 Department of Viral Vaccine, Chengdu Institute of Biological Products Co., Ltd, China National Biotech Group, Chengdu, China; 4 Centre for Vaccine Evaluation, Biologics and Genetic Therapies Directorate, Health Canada

## Abstract

In the course of isolating the attenuated Japanese encephalitis vaccine SA14-14-2, two attenuated strains SA14-9-7 and SA14-5-3 were also obtained that elicited low antibody responses in humans (<10% and 62%, respectively) and exerted much weaker immune protection in animal challenge experiments. However, the reason for these differences remains unknown. In order to understand why SA14-14-2 is superior to SA14-9-7 and SA14-5-3, we employed a reverse genetics method to identify the key mutations in the virus genome that determine the immunogenicity of live attenuated Japanese encephalitis viruses. We first sequenced the full genomic sequences of SA14-9-7 and SA14-5-3 and found mutations that changed four amino-acid base pairs when compared to the envelope gene of SA14-14-2. We mutated the genome of SA14-14-2 to generate these mutations both singly (E-177, E-264, E-279 and E-315) and in combination (E-177/264, E-279/315 and E-177/264/279/315) and tested these mutants along with parental strains SA14-14-2, SA14-9-7 and SA14-5-3 for their immunogenicity *in vivo*. When mice were immunized with SA14-9-7 and SA14-5-3, lower levels of neutralizing antibodies were generated compared with the immune response to SA14-14-2. Furthermore, SA14-5-3 was more immunogenic than SA14-9-7, which replicated the results previously seen in humans. Point mutations E-177, E-264, E-279 and E-315 diminished the immunogenicity of SA14-14-2 with E-264 and E-315, contributing the most to this phenotype. The mutant rJEV (E-177/E-264/E-279/E-315) containing all four point mutations exhibited the lowest immunogenicity with a seroconversion rate of 0 at an inoculation dose of 10^3^ PFU (plaque-forming unit). We have identified the key amino acids in the envelope protein that account for the superior immunogenicity of SA14-14-2.

## Introduction

Japanese encephalitis (JE) is a severe central nervous system disease, which is caused by infection with Japanese encephalitis virus (JEV). JEV is a mosquito-borne virus, mainly found in east and southeast Asia, but has gradually spread to other regions in recent years.^1^ Approximately one billion people are under the threat of JEV infection. The mortality rate of JEV infection can reach 30%, while up to 50% of survivors suffer permanent sequelae post recovery.^2^ Although there are no effective treatments, JEV infection can be prevented through controlling transmission by mosquitoes and vaccinating at-risk populations.

JEV belongs to the family *Flaviviridae* and is a mosquito-borne, enveloped virus. It has an ~11 kb positive sense single-stranded RNA genome that contains a 95-nt (nucleotide) 5ʹ untranslated region, a 586-nt 3ʹ non-translated region and a large open reading frame that encodes 3432 amino acids. This polyprotein comprises three structural proteins at its N-terminal region, including the capsid protein (C), pre-membrane protein (prM) and envelope protein (E). There are at least seven non-structural proteins to its C-terminal region, including NS1, NS2A, NS2B, NS3, NS4A, NS4B and NS5.

China suffers from a high rate of JE and experienced three major JE epidemics between the 1950s and 1970s. As a result of the JE vaccine vaccination program, the incidence of JEV infection has significantly declined in the past decade. The JE live vaccine SA14-14-2 is the most widely administered vaccine in China. Since its approval in 1989, more than 600 million doses of SA14-14-2 have been administered, protecting millions of children and markedly reduced the incidence and the mortality of JEV infection.^3^ The safety record of the SA14-14-2 vaccine has been extensively documented. It does not cause any illness when inoculated in mice or rhesus macaques, and no reversion to wild type has been observed when passaged in cells, mice or mosquitoes. The protective efficacy in humans is over 90%,^4–6^ and the SA14-14-2 vaccine has been licensed and used in many other countries including Korea, India, Nepal and others. On 9 October 2013, the SA14-14-2 vaccine passed the WHO pre-qualification and became the first such vaccine that was developed by China.^7^

The SA14-14-2 vaccine virus is a highly attenuated strain that was developed through serial passage of the JEV virulent SA14 strain in cells and animal non-neural tissues, followed by extensive and repeated plaque purifications. During this attenuation process, we also isolated two additional strains, SA14-9-7 and SA14-5-3, which elicited low levels of neutralizing antibodies and had poor protective efficacies in animal studies.^8,9^ The antibody seroconversion rate of SA14-9-7 is below 10%, while SA14-5-3 achieves 62% in humans. It remains unclear why SA14-14-2 is superior to SA14-9-7 and SA14-5-3 in terms of immunogenicity and induction of a protective immune response against virulent JEV. In order to better understand the underlying molecular mechanisms, we have sequenced the genomes of SA14-9-7 and SA14-5-3 and, relative to SA14-14-2, found several differences that resulted in changes at four amino acids in the viral E protein, a major determinant of viral virulence and viral immunogenicity. We next mutated these amino acids of SA14-14-2 and tested the neutralizing antibody response and protective efficacy induced in mice by these mutants.

## Materials and methods

### Sequencing SA14-5-3 and SA14-9-7

On the basis of the JEV sequence in GenBank, we designed seven pairs of primers to cover the entire genome of JEV (Table 1). All primers were synthesized by Shanghai Biotech Company.

### RNA extraction and full gene sequencing

Viral RNA was isolated from 100 μl JEV containing supernatants using an RNA isolation kit (Tiangen BioTec, Beijing, China). Reverse transcription of viral RNA was performed in a reaction mixture containing 5 μl of viral RNA, 5 μl of H_2_O, 1 μl of 10 mM dNTPs and 20 mM of downstream primer. RNA was first denatured at 65 °C for 5 min. After being chilled on ice, 4 μl of 5x buffer, 2 μl of 0.1 M dithiothreitol, 1 μl of RNase inhibitors and 1 μl of reverse transcriptase were added. Reverse transcription then proceeded at 37 °C for 50 min. Viral DNA was amplified in a mixture containing 1 μl of the reverse-transcribed products, 2.5 μl of 10× PCR buffer, 1 μl of each upstream and downstream primers, 0.5 μl of Pyrobest DNA polymerase and 19.5 μl of H_2_O. After denaturing at 94 °C for 5 min, 30 cycles of 30 s at 94 °C, 30 s at 55 °C and 120 s at 72 °C, with a final 10 min incubation at 72 °C, were conducted. The PCR products were harvested from 1% agarose gels after electrophoresis and sequenced at the Shanghai Biotech company. The sequences of SA14-5-3 and SA14-9-7 were aligned with that of SA14-14-2 using the NCBI Blast software (Bethesda, MD, USA).

### Construction of the JEV SA14-14-2 infectious clone

**Figure 1:**
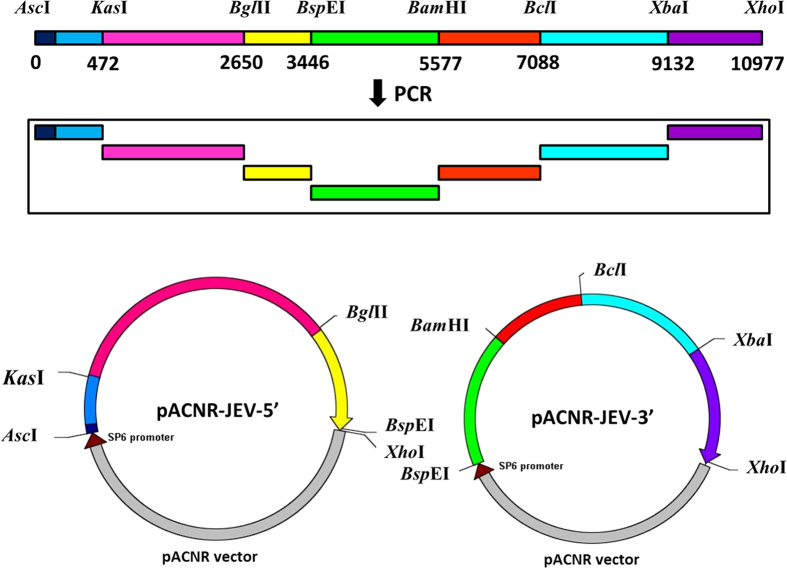
Construction of pACNR-JEV5ʹ and pACNR-JEV3ʹ plasmid. Seven fragments of JEV14-14-2 gene were obtained by PCR. The first three and the other four fragments were ligated and constructed into low-copy plasmid pACNR. The resulting plasmids were named pACNR-JEV5ʹ and pACNR-JEV3ʹ that contains JEV sequence from 1 to 3450 nt and from 3445 to 10 977 nt.

**Figure 2:**
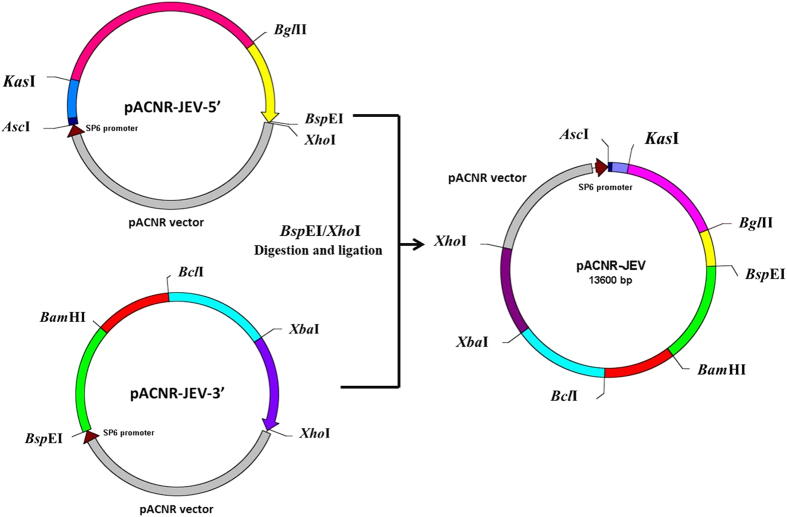
Construction of a plasmid that contains the full-length JEV genome. pACNR-JEV3ʹ was digested with *Bsp*EI and *Xho*I. The 7.5 kb DNA fragment was harvested and ligated into pACNR-JEV5ʹ, which was digested by the same restriction enzymes. The final plasmid DNA was named pACNR-JEV (1–10 977) that bears the complete JEV genome sequence.

Primers were first designed to amplify JEV gene in seven fragments using DNA polymerase PrimerSTAR (Takara; shown in Table 2). The fusion PCR was performed under the following conditions: first at 98 °C for 2 min, then 10 cycles at 98 °C for 10 s, 58.5–53.5 °C for 10 s, 72 °C (1 kb min^−1^), 20 cycles at 98 °C for 10 s, 53.5 °C for 10 s and 72 °C (1 kb min^−1^), followed by 10 min at 72 °C. The final PCR products were ligated into the T vector pMD19-T (Takara) and transformed into TOP10-competent bacteria, which were selected with ampicillin on an agar plate containing isopropyl-β-D-thiogalactoside and X-gal. The white colonies were picked for plasmid preparation. The correct clones (verified by sequencing) were used for ligating the correct JEV fragments into the low-copy plasmid pACNR. The first three fragments from the 5ʹ terminus (0.5, 2.2 and 0.8 kb) were sequentially cloned into pACNR using restriction enzymes *Asc*I/*Kas*I, *Kas*I/*Bgl*II and *Bgl*II/*Bsp*EI. The resulting plasmid was called pACNR-JEV5ʹ that contains JEV DNA from 1 to 3450 nt. The next four DNA fragments (2.1, 1.5, 2.1 and 1.8 kb) were sequentially cloned into pACNR using restriction enzymes *Bsp*EI/*Bam*HI, *Bam*HI/*Bcl*I, *Bcl*I/*Xba*I and *Xba*I/*Xho*I. The resulting plasmid was named pACNR-JEV3ʹ that contains JEV sequence from 3445 to 10 977 nt. This cloning strategy is illustrated in Figure 1. To construct a plasmid containing the complete JEV gene sequence, pACNR-JEV3ʹ was digested with *Bsp*E1 and *Xho*I. The 7.5 kb DNA fragment was harvested and ligated into pACNR-JEV5ʹ. The final plasmid DNA was named pACNR-JEV (1–10 977) that bears the complete JEV genome sequence (Figure 2).

### Generating JEV mutations

**Figure 3:**
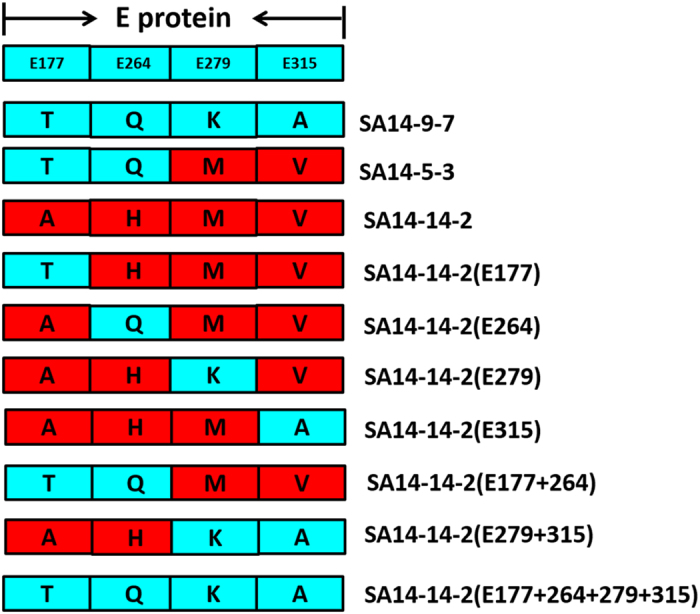
Amino-acid mutations of each new JEV strains. Compared with SA14-14-2, SA14-9-7 and SA14-5-3 had also stable attenuated neurovirulence but poor immunogenicity in human clinical trials. In order to study the mechanism of the different immunogenicity of SA14-14-2, the critical amino acids in E protein of SA14-14-2 have been mutated to counterparts of SA14-9-7 and SA14-5-3 both singly (E-177, E-264, E-279 and E-315) and in combination (E-177/264, E-279/315 and E-177/264/279/315). These mutant viruses named as rJEV(E-177), rJEV(E-264), rJEV(E-279), rJEV(E-315), rJEV(E-177/264), rJEV(E-279/315) and rJEV(E-177/264/279/315) were generated and then the immunogenicity of mutants investigated.

A PCR-based method was used to mutate the virus E gene in SA14-14-2 at positions E-177, E-264, E-279 and E-315 within pACNR-JEV5ʹ. The primers are listed in Table 3. The mutations were designed on the basis of the sequence alignment between SA14-14-2, SA14-5-3 and SA14-9-7. PCRs were performed with primers 177-F/R and 177-R/F (Table 3) under conditions of 98 °C for 30 s, 20 cycles of 98 °C for 10 s, 72 °C for 30 s and 72 °C for 1.5 min, and then 72 °C for 10 min. The PCR products were harvested, and then mixed and amplified for five cycles under 98 °C for 10 s, 72 °C for 30 s and 72 °C for 2.5 min. Primers F and R (Table 3) were then added into the reaction for 27 cycles of amplification under the above-mentioned conditions. The amplified DNA fragments were harvested, digested with *Kas*I and *Bgl*II and then ligated into pACNR-JEV5ʹ. The E-177 mutation was verified through sequencing. The 3ʹ fragment of JEV (7.5 kb) from pACNR-JEV3ʹ was obtained with digestion by *Bsp*EI and *Xho*I and ligated into pACNR-JEV5ʹ (E-177). The final DNA clone was named pACNR-JEV (E-177). The other mutations were engineered with the same strategy; they were named pACNR-JEV(E-264), pACNR-JEV(E-279), pACNR-JEV(E-315), pACNR-JEV(E-177/264), pACNR-JEV(E-279/315) and pACNR-JEV(E-177/264/279/315). Amino-acid mutations of each new JEV strains were show in Figure 3.

### Mutant virus recovery

Plasmid DNA containing the full-length JEV gene with the mutated gene was linearized with *Xho*I digestion. The digested DNA was harvested from agarose gels after electrophoresis, and was used to produce viral RNA using the Promega (Beijing, China) RiboMAX Large Scale RNA Production Systems-SP6. The *in vitro* synthesized JEV RNA was electroporated into BHK21 cells. Five days later, culture supernatants containing infectious JEV were harvested and designed as rJEV(E-177), rJEV(E-264), rJEV(E-279), rJEV(E-315), rJEV(E-177/264), rJEV(E-279/315) and rJEV(E-177/264/279/315).

### Virus identification and titer determination

After RNA extraction and complete gene sequencing to identify the recovered mutant virus, plaque assays were performed to determine the titers of recovered JEV. BHK21 cells were seeded into six-well plates to achieve monolayer of cells. Viruses were first diluted to 10^−3^, 10^−4^, 10^−5^ and 10^−6^, and then applied to BHK21 cell layers. Infections were performed in duplicates. After absorption for 1 h at 37 °C, media containing 1% methyl cellulose was added at 4 ml per well. Five days after culture at 37 °C, cells were stained with crystal violet. Virus plaques were counted and virus titers were calculated accordingly.

### Measuring the immunogenicity of JEV

We first monitored the production of neutralizing antibodies. The JEV tested in this study include SA14-14-2, SA14-5-3, SA14-9-7, rJEV(E-177), rJEV(E-264), rJEV(E-279), rJEV(E-315), rJEV(E-177/264), rJEV(E-279/315) and rJEV(E-177/264/279/315). These JEV strains were first prepared to 10^6^ and 10^4^ PFU ml^−1^, and then inoculated (subcutaneously, s.c.) into 10–12 g Kunming mice at a volume of 100 μl per mouse. For each JEV strain, 12 mice were inoculated at each dilution. The control group was treated with buffer. Fourteen days after immunization, blood was collected in each group and stored at 4 °C before being inactivated at 56 °C for 30 min. A Plaque Reduction Neutralization Test was performed to determine the titer of neutralizing antibodies. The highest dilution of serum that led to 50% reduction in virus plaques is designated as the neutralizing titer of this serum.

Animal protection assays were conducted as follows to determine protective efficacy of each of the viruses employed in this study. Mice were immunized (s.c.) at stated dilutions for each JEV strain. Control mice were treated with buffer. Fourteen days after immunization, 1000 LD50 of the virulent JEV strain P3 was used to infect mice intraperitoneally (i.p.) at a volume of 0.3 ml per mouse. Mice that died in 3 days were not counted. Fourteen days after JEV P3 challenge, surviving mice were scored. The ED50 values were inoculated for each JEV strain tested.

## Results

### Whole-genome analysis of attenuated JEV strains SA14-14-2, SA14-9-7 and SA14-5-3

We amplified the complete genomes of SA14-9-7 and SA14-5-3 in a series of DNA fragments, as detailed in the Materials and Methods. The sequencing results showed that all three strains contained 10 977 nucleotides, with a 95-nt 5ʹ non-translated region, a 586-nt 3ʹ non-translated region and a 3432-amino-acid open reading frame covering 96–10 391 nt. The highly attenuated strain SA14-14-2 shares 99.9% and 99.8% homology at the nucleotide level with SA14-5-3 and SA14-9-7, respectively, while SA14-9-7 and SA14-5-3 are 99.9% homologous. SA14-9-7 differs from SA14-14-2 at eleven amino-acid positions, four in the E protein (E-177, E-264, E-279 and E-315) and seven in the NS region. Six amino acids are different between SA14-5-3 and SA14-14-2, with two in the E protein (E-177 and E-264) and four in the NS region. Between SA14-9-7 and SA14-5-3, there are five amino-acid differences, two in the E protein (E-279 and E-315) and three in the NS region. Given the key role of E protein in the immunogenicity of JEV, we have focused our studies on the amino acids in the E protein.

### Mutations in the E protein diminish the ability of SA14-14-2 to elicit the production of neutralizing antibodies

**Figure 4:**
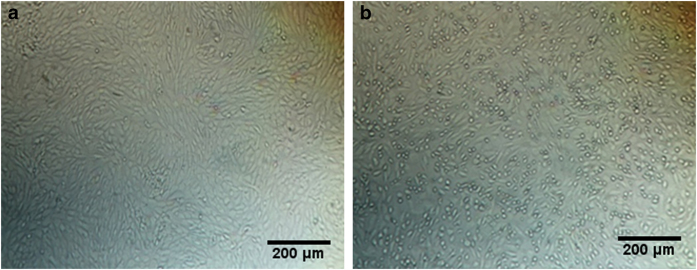
Cytopathic effect caused by mutant JEV. (**a**) Untransfected BKH21 cells; (**b**) transfected with rJEV(E-177) RNA. BHK21 cells were transfected with JEV RNA. Images shown are cells 5 days after transfection. Representative image of cells transfected with rJEV(E-177) RNA is shown.

In order to understand the molecular mechanism leading to the low immunogenicity of SA14-9-7 and SA14-5-3, we have investigated the possible role of the four amino-acid positions in the E protein that differ between these two JEV strains and SA14-14-2. We first changed the E protein amino acids at positions E-177, E-264, E-279 and E-315 in SA14-14-2 to their counterparts in SA14-9-7 or SA14-5-3 either individually or in different combinations, and generated mutations rJEV (E-177), rJEV (E-264), rJEV (E-279), rJEV (E-315), rJEV (E-177/264), rJEV (E-279/315) and rJEV (E-177/264/279/315). JEV viral RNA was synthesized *in vitro* and transfected into BHK21 cells to produce viruses. Five days after transfection, compared to control cells, the JEV RNA-transfected cells exhibited cytopathogenic effects (shown in Figure 4). Titers of viruses in the culture supernatants were determined in virus plaque assays, and all reached above 6.0  lg PFU ml^−1^. Sequences of the seven mutated viruses were also verified by RT-PCR and DNA sequencing; all contained the inserted mutations.

We then measured the abilities of these mutated JEV strains to elicit neutralizing antibodies when used to immunize mice. Two doses of each virus (10^3^ and 10^5^ PFU) were used in the immunization experiments. The results, as shown in Table 4, clearly demonstrate that the SA14-14-2 strain induced higher levels of neutralizing antibody production with better seroconversion rates compared to SA14-9-7 and SA14-5-3. Of the four E protein mutations tested, all diminished the immunogenicity of SA14-14-2, with E-264 and E-315 showing the greatest effect. When all four amino-acid mutations were changed into SA14-9-7, at the PFU dose of immunization, the seroconversion rate became 0. These data suggest that mutations E-264 and E-315 have a dominant role in reducing the immunogenicity of E protein, whereas mutations E-177 and E-279 have a less significant effect.

### Mutations in the E protein of SA14-14-2 reduce the immune protection capacity

We next measured the effect of the mutant viruses to protect mice from the virulent JEV strain P3. Mice were first immunized (s.c.) with different doses of SA14-14-2, SA14-5-3, SA14-9-7 and the other mutants. Fourteen days later, mice were infected with 1000 LD50 of JEV strain P3. Surviving mice were scored on day 14 after challenge. The results are summarized in Table 5. SA14-9-7 and SA14-5-3 showed markedly higher ED50 values of 2.89 and 2.8 compared to the 0.83 ED50 of SA14-14-2, which indicates that SA14-14-2 exerts a much stronger protection of mice from JEV P3 infection than the other two strains. When the SA14-14-2 mutants were tested, rJEV(E-177) and rJEV (E-264) had ED50 values of 1 and 1.11, and the ED50 values of rJEV (E-279) and rJEV (E-315) were 1.67 and 2.38, respectively. These data confirm our previous results indicating that all four mutations in the E protein negatively affect the ability of SA14-14-2 to provide protection against wild-type JEV, with the E-279 and E-315 mutations exerting the greatest effect.

## Discussion

In the process of isolating the attenuated JEV vaccine strain SA14-14-2, several attenuated strains were acquired, among which SA14-9-7 and SA14-5-3 were also tested by immunizing humans. It was found that SA14-9-7 elicited poor neutralizing antibody response in children with a seroconversion rate below 10%. SA14-5-3 was relatively more potent in this regard showing 63%. The capacity of SA14-14-2 to elicit neutralizing antibodies and a seroconversion rate of 92% was the best. We have now sequenced the whole genomes of SA14-9-7 and SA14-5-3 and found that, compared to SA14-14-2, SA14-9-7 had four mutations in E protein at positions E-177(A→T), E-264(H→Q), E-279(M→K) and E-315(V→A), and seven mutations in the NS region. Between SA14-9-7 and SA14-5-3, they differed at positions E-279 and E-315 in E protein, and NS1-339, NS2B-63 and NS5-386 in the NS region. SA14-9-7 and SA14-5-3 shared two common mutations in the E protein and four common mutations in the NS region compared to SA14-14-2.

As the E protein has a key role in JEV immunogenicity, we mutated the above four amino-acid positions in E protein in the context of SA14-14-2 and successfully generated seven viral mutants all of which reached a titer above 10^6^ PFU ml^−1^ in BHK21 cells. When a dose of 10^3^ PFU was used for immunization in mice, the seroconversion rates were 100, 33 and 0% for SA14-14-2, SA14-5-3 and SA14-9-7, respectively. A dose of 10^5^ PFU of the same three viruses resulted in seroconversion rates of 100, 83 and 66%. These results closely resemble the immune responses observed in humans when these strains were tested as vaccine candidates.

Among the four mutations tested, E-264(H→Q) and E-315(V→A) caused the greatest decrease in the production of neutralizing antibodies. Both mutations were found in SA14-9-7, whereas only E-264(H→Q) existed in SA14-5-3. The other two mutations E-177(A→T) and E-279(M→K) also markedly diminished the antibody response. The E-177(A→T) mutation existed in SA14-5-3, while both were present in SA14-9-7. The mutations E-177(A→T) and E-264(H→Q) were associated with the lowered immunogenicity of SA14-5-3. However, when all four mutations were combined, the level of neutralizing antibody response was seriously compromised, similar to the phenotype observed for SA14-9-7.

In the murine challenge experiments, SA14-14-2 induced a robust protection from the JEV P3 strain compared to SA14-9-7 and SA14-5-3. All mutated strains showed diminished protective capacity compared to the parental strain SA14-14-2; yet the degree of reduction was not as great as the decrease in neutralizing antibodies. This difference may be the result of the fact that the mutated SA14-14-2 strains bear all the parental strain NS genes. Although the NS gene products of flaviviruses do not stimulate the production of neutralizing antibodies, they do confer a protective effect.^10–14^ In our previous studies, we observed that spleen cells that were immunized with SA14-14-2 effectively protected mice from virus infection, even though mice had a low level of neutralizing antibodies (<10).^15^ Similar findings were made with guinea pigs that had low levels of neutralizing antibodies and yet survived challenge from virulent strains.^16^ It has also been reported that immunization with SA14-14-2 NS1 protein induced a strong protective response in mice.^17^

In agreement with the role of NS gene products in immune protection, sequence analysis has shown that SA14-9-7 has seven mutations in the NS region, with two in NS1, one in NS2B, one in NS3 and three in NS5 compared to SA14-14-2. Four of these mutations (one in NS1, one in NS3 and two in NS5) are shared by SA14-5-3; the other three are specific to SA14-9-7 including NS1-339, NS2B-63 and NS5-386. These may also contribute to the lowered immunogenic capacities of these two JEV strains. As SA14-9-7 induces a lesser protective response than SA14-5-3, we speculate that, in addition to the difference in their E proteins, the three NS mutations that are specific to SA14-9-7 may also have a role, although this will require further investigation.

In summary, for the first time we have reported the molecular mechanism that underlies the different immunogenicity of three attenuated JEV strains SA14-14-2, SA14-5-3 and SA14-9-7. Our data demonstrate the important role of four specific amino-acid positions in the E protein, E-177, E-264, E-279 and E-315, in modulating neutralizing antibody response and immune protection efficiency of JEV vaccine strains. These results shed further lights on the immunization mechanism of JEV and other flaviviruses, and are expected to guide the development of new flavivirus vaccines. Furthermore, this study will help us to improve the quality-control level to control the efficacy of JE-attenuated live vaccine SA14-14-2.

## Human and animal rights, and informed consent

All institutional and national guidelines for the care and use of laboratory animals were followed.

## Figures and Tables

**Table 1:** Primers to sequence JEV

*Primers*	*Sequences*	*Positions (nt)*
P1	P1L:5′- AGAAGTTTATCTGTGTGAACTTCTTGG-3′	1–27
	P1R:5′- TATCGAAGGAGCATTGGG-3′	1488–1505
P2	P2L:5′- GAGAAGCCCACAACGAGAAG-3′	1210–1229
	P2R:5′- GAATCGTAGGGGCGGAAG 3′	3428–3446
P3	P3L:5′- GAACTCATCATTCCGCATACCATAG-3′	3195–3220
	P3R:5′- CCATTTTCGGTCAAACCTCC-3′	4855–4874
P4	P4L:5′- TAGCCGCCCTCACGCCTTG-3′	4534–4552
	P4R:5′- CAGTGCATCTGGCAGCTCTTCG-3′	6596–6617
P5	P5L:5′- GGAAAATCCTCAAGCCGAGAT-3′	6373–6393
	P5R:5′- TCAGGAGCCTTCGTGTCAACT-3′	8706–8726
P6	P6L:5′- CAGGTACTACTGGGGCGAATG-3′	8388–8408
	P6R:5′- TCCTCTGCACGGGACAACTAT-3′	9861–9881
P7	P7L:5′- AAGACCGTGATGGACGTGATATC-3′	9444–9466
	P7R:5′- AGATCCTGTGTTCTTCCTCACC-3′	10 952–10 977

Abbreviations: JEV, Japanese encephalitis virus; nt, nucleotide.

**Table 3:** Primers used to generate JEV mutations

*Primers*	*Sequences*	*Mutations*
177-F	5′- CCAATGCTCCTTCGGTA**A**CCCTCAAAC-3′	G to A
177-R	5′- GTTTGAGGG**T**TACCGAAGGAGCATTGG-3′	C to T
264-F	5′- GAAGGAGGCCTCCATCA**G**GCGTTG-3′	T to G
264-R	5′- CAACGC**C**TGATGGAGGCCTCCTTC-3′	A to C
279-F	5′ - GTACTCAAGCTCAGTGA**A**GTTAACATCAGG-3′	T to A
279-R	5′- CCTGATGTTAAC**T**TCACTGAGCTTGAGTAC-3′	A to T
315-F	5′- GAAAAATCCGG**C**GGACACTGGTCAC-3′	T to C
315-R	5′- GTGACCAGTGTCC**G**CCGGATTTTTC-3′	A to G
F	5′- TTGGCGCGCCAATGCAGG**C**GCCATGAAGTTGTCGAAT-3′, *Asc*I, *Kas*I	A to C silent mutation
R	5′- GGTCCGGACCAGTCTAGTGACAGATCTGACTC-3′, *Bsp*EI, *Bgl*II	

Abbreviation: JEV, Japanese encephalitis virus. Bold is the location of the mutation. The underline entities are the restriction enzyme sites.

**Table 4:** Titers of neutralizing antibodies elicited by mutated JEV in mice

*Viral strains*	*Seroconversion (%)*		*GMT*	
	*10*^*3*^ *PFU*	*10*^*5*^ *PFU*	*10*^*3*^ *PFU*	*10*^*5*^ *PFU*
SA14-14-2	100	100	28.3	50.4
SA14-5-3	33.3	83.3	2.2	17.2
rJEV (E-177)	66.7	83	4.6	24.3
rJEV (E-264)	16.7	50	1.6	4
rJEV (177/264)	50	83	3.2	30.6
SA14-9-7	0	66.7	0	8.3
rJEV (E-279)	66.7	100	7.4	44.9
rJEV (E-315)	16.7	83.3	2.1	7.6
rJEV (E-279/315)	66.7	33.3	9.3	2.7
rJEV (E-177/264/279/315)	0	33.3	0	3

Abbreviations: GMT, geometric mean titer; JEV, Japanese encephalitis virus; PFU, plaque-forming unit.

**Table 5:** Protection capacity of attenuated JEV strains

*Viral strains*	*Route*	*Dose (PFU)*	*Mortality no. dead/no. tested*	*ED50 (l g PFU)*
SA14-14-2	s.c.	10	3/9	0.83
	s.c.	100	1/10	
	s.c.	1000	0/10	
SA14-5-3	s.c.	10	8/10	2.8
	s.c.	100	8/10	
	s.c.	1000	6/10	
rJEV (E-177)	s.c.	10	5/10	1
	s.c.	100	0/10	
	s.c.	1000	0/10	
rJEV (E-264)	s.c.	10	4/10	1.11
	s.c.	100	3/10	
	s.c.	1000	0/10	
rJEV (E-177/264)	s.c.	10	3/10	0.86
	s.c.	100	2/10	
	s.c	1000	0/10	
SA14-9-7	s.c.	10	8/10	2.89
	s.c.	100	8/9	
	s.c.	1000	6/10	
rJEV (E-279)	s.c.	10	6/10	1.67
	s.c.	100	4/10	
	s.c.	1000	2/10	
rJEV (E-315)	s.c.	10	8/10	2.38
	s.c.	100	8/10	
	s.c.	1000	2/10	
rJEV (E-279/315)	s.c.	10	8/10	2.28
	s.c.	100	7/10	
	s.c.	1000	2/10	
rJEV (E-177/264/279/315)	s.c.	10	9/10	1.84
	s.c.	100	4/10	
	s.c.	1000	1/10	

Abbreviations: JEV, Japanese encephalitis virus; PFU, plaque-forming unit; s.c., subcutaneous.

**Table 2:** Primers for constructing JEV-infectious DNA clone

*Primers*	*Sequences*
F1	5′- TTGGCGCGCCATTTAGGTGACACTATAGAGAAGTTTATCTGTGTGAACTTCTT-3′, *Asc*I sp6 promoter
R1	5′- CCTGG**C**GCCTGCACAAGCTATGACAAC-3′, *Kas*I
F2	5′- TTGGCGCGCCAATGCAGG**C**GCCATGAAGTTGTCGAAT-3′, *Asc*I, *Kas*I
R3	5′- GGTCCGGACCAGTCTAGTGACAGATCTGACTC-3′, *Bsp*EI, *Bgl*II
F4	5′- TGCGGAGTCAGATCTGTCACT-3′, *Bgl*II
R4	5′- CATTTTCTGTCCGGAATCGTAG-3′, *Bsp*EI
F5	5′- CGCCCCTACGATTCCGGACAGA-3′, *Bsp*EI
R6	5′- GGAAAAGGATCCGTGGTTCCAGG-3′, *Bam*HI
F7	5′- ACCACGGATCCTTTTCCTGACTC-3′, *Bam*HI
R8	5′- CCGCTCGAGCGGTGCTCTAGAACGCGTGTATTCCGACGTGATCAGGTG-3′, *Xho*I, *Xba*I, *Mlu*I, *Bcl*I
F9	5′- CACCTGATCACGTCGGAATA-3′, *Bcl*I
R10	5′- CCGCTCGAGCCCAAAGCTTCAAACTCTAGAT-3′, *Xho*I, *Xba*I
F11	5′- GCTTGGAGCACGGTATCTAGAGTT -3′, *Xba*I
R11	5′- CCGCTCGAGAGATCCTGTGTTCTTCCTCACCAC-3′, *Xho*I

*Serial nos*	*Mutations*	*SA14-14*	*SA14-14-2*
1	E-107	Phenylalanine	Leucine
2	E-138	Lysine	Phenylalanine
3	E-176	Valine	Isoleucine
4	E-177	Alanine	Threonine
5	E-264	Methionine	Lysine
6	E-279	Valine	Alanine
7	E-315	Proline	Serine

Abbreviation: JEV, Japanese encephalitis virus. Underline entities are the restriction enzyme.
